# Improved Stability and In Vitro Anti-Arthritis Bioactivity of Curcumin–Casein Nanoparticles by Ultrasound-Driven Encapsulation

**DOI:** 10.3390/nu14235192

**Published:** 2022-12-06

**Authors:** Kexin Li, Yahui Zhang, Xueyan Hao, Dongchao Xie, Chongchong Wang, Haihua Zhang, Peng Jin, Qizhen Du

**Affiliations:** College of Food and Health, Zhejiang Agriculture and Forestry University, No. 666 Wusu Road, Linan District, Hangzhou 311300, China

**Keywords:** sodium caseinate, ultrasound treatment, curcumin, nanoparticles, arthritis

## Abstract

Curcumin possesses beneficial biological functions, namely anti-inflammation and anti-diabetic functions. However, due to its low solubility and crystallinity, its applications are limited. In this work, curcumin was encapsulated in casein micelles in order to form curcumin-casein nanoparticles by ultrasound treatment (5 min). The ultrasound treatment induced the entry of the hydrophobic groups to the inner micelles and the polar sulfydryl groups to the surface of the micelles in order to form compact curcumin-casein nanoparticles of an appropriate size (100–120 nm) for cellular endocytosis. The product exhibited excellent stability during 8 months of cold storage, 6 days at room temperature, and 2 days at body temperature. Advanced in vitro experiments demonstrated that curcumin-casein nanoparticles displayed significantly greater inhibitory activity against the proliferation and proinflammatory cytokines of human fibroblast-like synoviocyte-osteo arthritis (HFLS-OA) cells and HFLS-rheumatoid (RA) cells than native curcumin due to better cellular uptake as a result of the low crystallinity and the appropriate nano-size of the nano-form. The results provide a reference for the use of ultrasound treatment to encapsulate other drug molecules and curcumin-casein nanoparticles as potential treatment for arthritis.

## 1. Introduction

Curcumin is the primary polyphenol in the curcuminoid family of turmeric, a prominent ginger family spice. Curcumin has a wide range of beneficial biological activities, namely anti-inflammatory, anti-diabetic, antibacterial, and anti-cancer properties, aside from its traditional use as an antioxidant and a food coloring. However, curcumin has drawbacks, such as a short shelf life due to its low stability, poor bioavailability due to its low water solubility and poor absorption, quick systemic elimination, and quick metabolism. Nanoencapsulation is an emerging strategy that is employed in order to resolve these drawbacks. For the loading of curcumin, several delivery systems have been employed, including nature-inspired, polymer- and biopolymer-based, lipid-based, surfactant-based, and special equipment-based methods [1].

Curcumin that is encapsulated in nano-emulsions has higher dispersibility than raw curcumin [2]. Liposomes can efficiently incorporate curcumin into their phospholipid bilayers, increasing cellular absorption whilst shielding the drug against chemical degradation and photodegradation [3], thereby enhancing the water solubility and bioavailability of curcumin [4]. Solid lipid nanoparticles (SLNs) may improve the stability of curcumin during preservation at ambient and refrigerated temperatures [5] and may sustain curcumin release with better dispersibility in aqueous-based foods [6]. Curcumin has been encapsulated into polymeric nano-micelles with a hydrophilic shell and a hydrophobic core [7]. Curcumin that has been loaded in conjugate proteins and polysaccharide nanoparticles has been shown to have smaller particle sizes, higher stability, and increased release under simulated gastrointestinal environments [8]. Ning et al. 2018 [9] encapsulated almost 100% of curcumin in covalently cross-linked injectable hydrogels based on poly(ethylene glycol) diacrylate and thiolated chitosan; the product efficiently inhibited tumor growth in vivo. The above encapsulation of curcumin into nano-carriers may be a possible solution to overcome its inherent limitations. However, there remain reasonable concerns about industrialization feasibility and toxicological safety when the nanoparticles of curcumin enter the biological pathway. Therefore, the nanoencapsulation of curcumin with food-grade materials in order to establish curcumin nanoparticles with sufficient stability and adaptability to industrialization remains challenging [10].

Caseins, the main protein constituent of bovine milk, are a commercially accessible food-grade additive in beverages and food [11]. Caseins are remarkably stable at high pressures and temperatures in their native state (i.e, treated at 100 MPa and 100 °C without compromising their fundamental integrity). Natural caseins can self-assemble into a micellar structure with diameters ranging from 10 to 400 nm [12]. Since caseins are amphiphilic, they behave more like block copolymers with alternating charge and hydrophobicity, which are excellent for incorporating substances with low water solubility in the micelle’s hydrophobic core [13]. However, caseins are frequently used as effective nanocarriers for the sustained release of hydrophobic drugs [14].

Casein nanoparticles have also been encapsulated with curcumin. Sahu et al. [15] encapsulated curcumin into bovine casein micelles based on the self-assembly abilities of the protein. Native-like phosphocaseins might supply a functional nano-system [16]. By spray drying a hot solution of aqueous ethanol containing co-dissolved sodium caseinate and curcumin, Pan et al. [17] developed a novel encapsulating technique. Pan et al. [18] also synthesized curcumin incorporated by caseinate by using the self-assembly characteristics of sodium caseinate and the pH-dependent solubility characteristics of curcumin. A hierarchical encapsulation approach, utilizing the ligand binding of curcumin to sodium caseinate, formed curcumin–casein nanoparticles [19]. However, the encapsulation of curcumin into casein nanoparticles could be a novel method to improve its crystallinity and bioavailability [20].

Ultrasound-assisted or sonication strategies have emerged as possible candidates in numerous processes/industries. However, the heat generated by an ultrasound affects the properties of the materials. Sylia et al. [21] discussed the importance of structural modification and its physical features in many applications. Farid et al. [22] described the use of ultrasound-assisted methods in food industries. Higuera–Barraza et al. [23] investigated the impact of ultrasonication and other factors on protein functional characteristics. The ultrasound-assisted approach is a green synthesis process that finds new applications in the food industry because of its low environmental effects and its high hygienic qualities. In this work, we developed an ultrasound-assisted technique to modify the physicochemical properties of casein micelles and to obtain curcumin–casein nanoparticles with low crystallinity and enhanced bioavailability.

## 2. Materials and Methods

### 2.1. Reagents

Curcumin (>99.0%) was acquired by Taizhou Crene Biotechnology Co., Ltd., (Taizhou, China). CCK-8 and sodium casein (90%) were acquired from Sigma–Aldrich Corp. (St. Louis, MO, USA). Tumor necrosis factor α (TNF-α) and the enzyme-linked immunosorbent assay (ELISA) kits for human vascular endothelial growth factor, human TNF-α, human interleukin-2 like (IL-2L), human interleukin-6 (IL-6), and human matrix metalloproteinase 1 (MMP-1) were acquired from Sigma–Aldrich (Shanghai, China). The other reagents utilized were high-performance liquid chromatography (HPLC), biological, and analytical grades.

### 2.2. Arthritis Cells

Human fibroblast-like synoviocyte–rheumatoid arthritis cells (HFLS-RA) and human fibroblast-like synoviocyte–osteoarthritis cells (HFLS-OA) were acquired from Shanghai Cells Bank, Institute of Life Sciences, Chinese Academy of Sciences (Shanghai, China).

### 2.3. Ultrasound Treatment for Sodium Casienate

Sodium caseinate was fully dissolved in water and four solutions of 10, 20, 30, and 40 mg/mL concentrations were prepared. Each caseinate solution (20 mL) was placed in a 50 mL tube and ultrasonicated (500 W, 5 s on and 1 s off) using a SCIENTZ-950E Ultrasonic processor (Ningbo Scientz Biotechnology Co., Ltd., Ningbo, China). The tube was placed in an ice bath in order to control the temperature during ultrasonication. After the ultrasonication, the caseinate dispersion was filtered using a 460 nm film for the advanced measurement of size, zeta potential, polydispersion index (PDI), surface free sulfydryl and total free sulfydryl, and the surface hydrophobicity of the casein micelles.

### 2.4. Characterization of Casein Micelles

At 25 ± 0.1 °C and a scattering angle of 90°, the size of the nanoparticles was studied utilizing a zeta potential measurement (Zetasizer ZSE, Malvern Instruments, Malvern, UK). The volume measurement was used to assess particle size distribution, which determines the proportional percentage of particles in each size category based on the volume they cover. Laser Doppler velocimetry (LDV) was used in order to determine the zeta potential utilizing the same Zetasizer Nano-ZSE analyzer (Malvern Instruments, Malvern, UK).

### 2.5. Estimation of Free Sulfhydryl Group Contents

The content of the free sulfhydryl groups (SH) of the casein micelle samples containing free SH was determined using Ellman’s reagent (5′,5-dithiobis (2-nitrobenzoic acid), DTNB) [24]. Briefly, 0.5 mL of the casein micelle sample was mixed with 2.5 mL of 8 M urea in a tris-glycine buffer (10.4 g of Tris, 6.9 g of glycine, 1.2 g of ethylenediaminetetraacetic acid (EDTA) per liter, pH 8.0) and 0.02 mL of Ellman’s reagent (4 mg/mL DTNB in tris-glycine buffer) for a color reaction. A UV-1800 spectrophotometer (Shimadzu Kyoto, Japan) measured the absorbance at 412 nm after 15 min. Using the following equation, the content of free SH (µM SH/g) was determined:

μM SH⁄g = (73.53 × A_412_ × D)/C

where D denotes the dilution factor, A_412_ denotes the absorbance at 412 nm, C denotes the casein micelle dispersion content (mg/mL), the factor 73.53 was obtained from 10^6^/(1.36 × 10^4^), and 1.36 × 10^4^ denotes the molar Ellman absorptivity constant.

### 2.6. Determination of Surface Hydrophobicity

The surface hydrophobicity of the casein micelle samples was assessed based on the approach used by Wang et al. [25]. In order to achieve various protein concentrations (0.02–2 mg/mL), dilutions of 2% (*w*/*v*) casein micelle dispersions were produced using phosphate buffer (pH 6.8, 50 mM). An amount of 0.6 mL of 8-anilino-1-naphthalene sulfonic acid (ANS, pH 6.8, 8 mM in 50 mM phosphate buffer) was dissolved in 10 mL of all the dilutions and was allowed to react for 15 min. An F-7000 fluorescence detector (Hitachi Scientific Instruments Co., Ltd., Beijing, China) was utilized in order to analyze the fluorescence of each sample. The analysis was conducted with excitation and emission at 390 nm and 480 nm, respectively. The initial slope of the relative fluorescent intensity (RFI) versus the protein content of the serial dilutions was used to compute the solutions’ surface hydrophobicity index (SHI). RFI was described by RFI = (F − F_0_)/F_0_, where F_0_ is the reading of the ANS solution without casein and F is the fluorescence reading of the protein–ANS conjugate.

### 2.7. Preparation of Curcumin–Casein Nanoparticles

Sodium caseinate solutions of 10, 20, 30, and 40 mg/mL concentrations were obtained by completely dissolving the appropriate amounts of sodium caseinate in water, and curcumin solution with a concentration of 20 mg/mL was prepared with ethanol as solvent. The curcumin solution was dissolved in the sodium caseinate solution in a proportion of 1:10 (curcumin: caseinate, *w*/*w*). The mixture was ultrasonicated for 2.5, 5, 7.5, and 10 min. The denatured protein was eliminated by filtering with a 460 nm film after ultrasonication. Finally, a curcumin–casein nanoparticle (Cur-CS-NPs) dispersion was obtained. The curcumin amount in the Cur-CS-NPs dispersion was fully regarded as encapsulated curcumin because the non-encapsulated curcumin was removed by filtration.

### 2.8. Loading Content and Encapsulation Efficiency (EE)

In previous EE determinations, the Cur-CS-NPs dispersion was filtered using 460 nm film. The filtrate was shifted and combined with many volumes of chloroform (to the range of the calibration curve). The bottom chloroform phase of the mixture was shifted and analyzed for absorbance at 419 nm (OD419) utilizing a UV-1800 spectrophotometer (Shimadzu, Kyoto, Japan) after being stirred overnight at room temperature (22 °C) and after the mixture split into two phases. Based on the calibration curve created previously using standard solutions containing various concentrations of free curcumin dissolved in chloroform, OD419 was employed to calculate the curcumin concentration [18]. The EE was determined as the % of curcumin in the filtrate relative to the total curcumin utilized in the incorporation. The % of the weight of curcumin relative to the combined weight of curcumin and sodium caseinate was used to evaluate the loading capacity. Three replicates were evaluated independently.

### 2.9. Characterization of Nanoparticles

Zeta potential measurements (Zetasizer ZSE, Malvern Instruments, Malvern, UK) were used in order to assess the size of the nanoparticles (NPs). Transmission electron microscopy (H-9500E, Hitachi, Tokyo, Japan) was used in order to evaluate the size and morphology of the NPs in further detail. A diluted NP dispersion was adsorbed onto a carbon-coated Formvar film connected to a metal specimen grid. Blotting was used to remove the excess sample, and the grid was enveloped with a tiny drop of staining solution (2% *w*/*v* phosphotungstic acid). After a short period, the excess staining solution was removed from the grid. After the material had properly dried by air, a transmission electron microscope was used to analyze it.

### 2.10. X-ray Diffraction (XRD)

An X-ray diffractometer (X’Pert-Pro MPD, PANalytical, Almelo, The Netherlands) with copper (Cu) Kα radiation was employed in order to determine the crystal structures of dried Cur-CS-NPs from the lyophilization of the Cur-CS-NPs dispersions. At 6°/min, 30 mA, and 40 kV, XRD data were received in the range of 2θ from 5° to 60°.

### 2.11. Storage Stability Assay of Cur-CS-NPs

The Cur-CS-NPs dispersions that were produced were stored for predetermined times at 4 °C (2, 4, 6, 8, and 10 months), 25 °C (2, 4, 6, and 8 days), and 37 °C (1, 2, 3, and 4 days). The PDI, size, and zeta potential of the Cur-CS-NPs were measured.

### 2.12. Viability Determination of Arthritic Cells In Vitro

In order to examine arthritis inhibition in vitro, the results of all the produced samples on the viability of HFLS-RA and HFLS-OA were investigated [26]. In a cell incubator set at 37 °C and 5% carbon dioxide (CO_2_), HFLS-RA cells and HFLS-OA cells were grown in a high-sugar Dulbecco’s modified eagle medium (DMEM) combined with 10% fetal bovine serum and 1% penicillin. At 37 °C, the cells were kept in humidified monolayer cultures in a 5% CO_2_ and 95% air environment. They were used in tests when the cell growth achieved around 70% confluence. Trypsin was used to break down the cell suspension, diluting the solution to the necessary concentration. For 24 h, the cells were injected onto 96-well plates (200 μL/well). Once the cells had attached to the wells and reached a confluency of about 70%, 20 μL of the drug at a specific amount was introduced into the wells. An amount of 10 μL of the CCK-8 reagent was introduced during the reaction’s first hour after the cells had been grown in the incubator for another 48 h. Using a Bio-Rad microplate reader (BioTek, Winooski, VT, USA) and a reference sample as a blank, absorbance at 450 nm was measured.

Cell viability was determined by the equation given below:

Cell viability (%) = (*A*_treated_/*A*_control_) × 100

where *A*_control_ and *A*_treated_ are the absorbance of the untreated and treated cells, respectively.

### 2.13. Enzyme-Linked Immunosorbent Assay

HFLS-RA and HFLS-OA cells (6 × 10^4^ cells/well) were seeded in 96-well plates and incubated for 48 h with Cur-CS-NPs and native curcumin (curcumin 40 μg/mL in the initial culture medium) in the presence of tumor necrosis factor-α (TNF-α, 10 ng/mL). Commercial ELISA kits (R&D, Minneapolis, MN, USA) were used to quantify the levels of interleukin IL-8, IL-6, IL-1β, and MMP-1 in the cell supernatant as per the manufacturer’s guidelines [27].

### 2.14. In Vitro Cell Uptake Studies

HFLS-OA and HFLS-RA cells were used in a practical method to conduct curcumin cell uptake experiments. HFLS-OA cells or HFLS-RA cells (5 × 10^5^ cells/mL) were plated in 24-well Nunclon Delta cell culture plates (Nunc, Wiesbaden, Germany) filled with RPMI 1640 media comprising 1% antibiotics and 10% heat-inactivated fetal bovine serum (Gibco, Thermo Scientific, Waltham, MA, USA). The adherent cells were treated with curcumin and Cur-CS-NPs (equivalent to 40 μg/mL curcumin in the final culture medium) after 6 h of incubation (37 °C/5% CO_2_), reconstituted in RPMI 1640 medium, and incubated for 24 h in a similar environment. The culture medium and non-adherent cells were isolated after 12, 24, 36, and 48 h, and the adherent cells were then rinsed three times with fresh water. The adhering cells were removed using a scraper. The obtained cells were dispersed in 10 mL of ethanol and processed with an ultrasonic cell pulverizer in order to isolate curcumin. Centrifugation (10,000× *g*) was used to isolate the pulverized cell residues and the extraction solution, and the supernatant was then applied to ultraperformance liquid chromatography mass spectrometry (UPLC-MS/MS) (Waters, Milford, MA, USA) in order to assess the intracellular curcumin. The UPLC-MS/MS analysis was carried out according to the procedure described by Wu et al. [28]. The UPLC system included a Waters XEVO TQD triple-quadrupole with an electrospray ionization source and a BEH C18 column (2.1 mm × 50 mm, 1.7 μm), as well as a binary solvent manager and a sample manager with a flow-through needle. For data collection and instrument control, Masslynx 4.1 software (Waters Corporation, Milford, MA, USA) was utilized. The mobile phase was made up of 0.1% formic acid (A) and methanol (B) with a gradient: 40% (A) and 60% (B) from 0 to 1.0 min, 40–10% (A) and 60–90% (B) from 1.0 to 3.0 min, 10–40% (A) and 90–60% (B) from 3.0 to 3.5 min, and 40% (A) and 60% (B) from 3.5 to 4.0 min at a flow rate of 0.4 mL/min. Curcumin was measured in a positive ion mode utilizing the multiple reaction monitoring (MRM) method. For the ion mass spectrometric study of curcumin *m*/*z* 367→149, the collision voltage and the cone voltage were set at 20 V and 60 V, respectively.

### 2.15. Statistical Analyses

For all the groups, data are reported as means and standard deviations (means ± standard deviation (SD)). Dunnett’s post-hoc test was used after a one-way analysis of variance (SPSS software version 19.0, SPSS Inc., Chicago, IL, USA) in order to identify statistically significant differences between the groups. *p* < 0.05 was used to determine significant differences.

## 3. Results and Discussion

### 3.1. Change in Casein Micelle Properties Driven by Ultrasound Treatment

**Figure 1:**
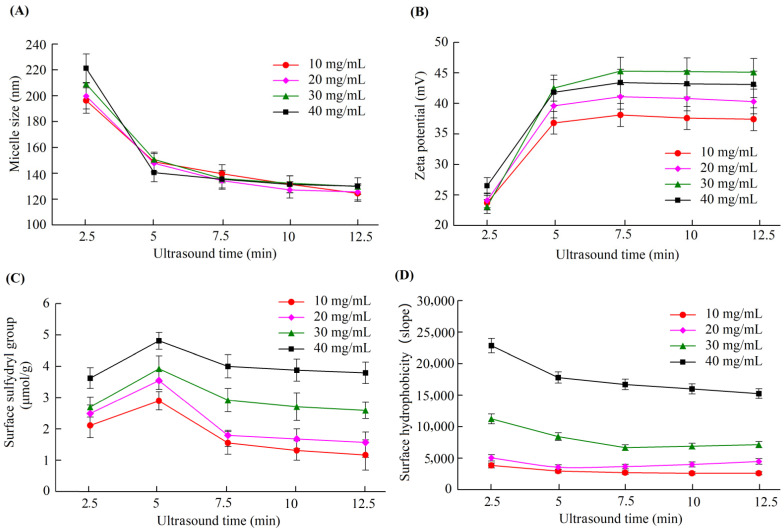
Changes in the size (**A**), zeta potential (**B**), surface free sulfhydryl group (**C**), and surface hydrophobicity (**D**) of casein micelles formed by ultrasound treatment of various caseinate concentrations with various times.

We performed ultrasound treatment for different caseinate solutions and studied the influence of ultrasound treatment on the properties of the micelles. Figure 1 shows the variations in the micelle zeta potential, size, surface free sulfhydryl, and the surface hydrophobicity of the casein micelles formed from caseinate solutions with concentrations of 10, 20, 30, and 40 mg/mL driven by ultrasound treatment for different times. When the caseinate solution was ultrasound treated for 5 min, the micelle size and the surface hydrophobicity significantly decreased, whereas the zeta potential and the surface free sulfhydryl groups significantly increased compared with ultrasound treatment for 2.5 min. Casein proteins assemble into casein micelles mainly due to intermolecular hydrophobic interactions. After the appropriate ultrasound treatment, the casein proteins rearranged to make more polar groups on one side and more nonpolar groups on the other side. Consequently, the amount of surface free sulfhydryls and the inner hydrophobicity of the formed casein micelles increased. Therefore, the casein micelles led to a smaller size and a higher zeta potential. However, ultrasound treatment for 7.5, 10, and 12.5 min did not yield significant changes in the zeta potential, size, surface free sulfhydryls, nor the surface hydrophobicity of the casein micelles. Hence, an ultrasound treatment time of 5 min is the best to induce the casein proteins to assemble into the most stable casein micelles. Ultrasound treatment from 2 to 6 min was reported to increase the intrinsic fluorescence intensities of the casein samples [29], leading to enhanced hydrophobic interactions between the tyrosine residues and the inner micelles and decreased surface hydrophobicity. Meanwhile, exposure to the micellar surface increased the number of surface sulfydryls. These results support the ultrasound-induced changes in the properties of casein micelles.

### 3.2. Formation of Curcumin Nanoparticles Driven by Ultrasound Treatment

**Figure 2:**
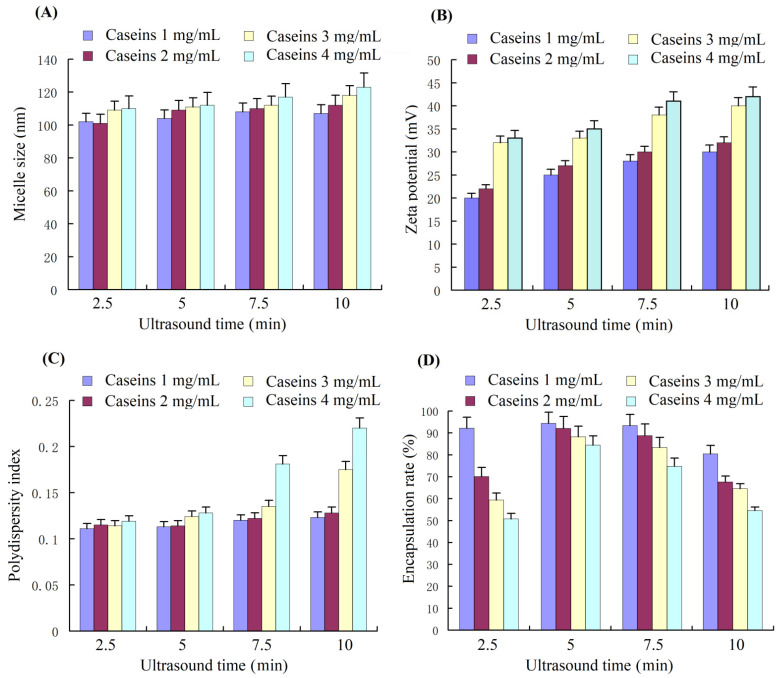
The average particle size (**A**), zeta potential (**B**), PDI (**C**), and curcumin encapsulation rate (**D**) of curcumin–casein nanoparticles assembled with a 1:1 ratio of curcumin/caseinate (*m*/*m*) with different caseinate concentrations driven by ultrasound treatment with different times. PDI, polydispersion index.

We prepared curcumin nanoparticles (curcumin NPs) by ultrasound treatment, which could change the size, zeta potential, surface free sulfhydryl group, and surface hydrophobicity of the casein micelles. Ultrasound treatment influenced the size, zeta potential, PDI, and encapsulation of curcumin (Figure 2). The average nanoparticle size ranged from 100 nm to 120 nm for all the four caseinate solutions under 2.5, 5, 7.5, and 10 min of ultrasound treatment (Figure 2A). Hence, we infer that the hydrophobicity of curcumin is beneficial for curcumin nanoparticles due to the hydrophobic interaction between curcumin and the hydrophobic residues of the casein protein inside the nanoparticles. The zeta potential values of the curcumin nanoparticles that were formed by ultrasound treatment for 2.5, 5, 7.5, and 10 min (Figure 2A) were similar to those of the casein micelles yielded by corresponding ultrasound treatment times (Figure 1B). Hence, curcumin had an indiscernible influence on zeta potential. Only ultrasound treatment for 7.5 and 10 min for the high caseinate concentrations (3 and 4 mg/mL) were significantly different from the other samples (Figure 2C). Regarding the curcumin encapsulation of the curcumin nanoparticles, the lowest caseinate concentration exhibited the highest encapsulation rate. Ultrasound treatment for 5 min led to the best encapsulation (>90%) compared with ultrasound treatment for 2.5, 7.5, and 10 min. This finding indicated that an appropriate ultrasound duration was needed to drive all the curcumin to the nanoparticles, whereas excessive ultrasound treatment could lead to the leaking of curcumin from the curcumin nanoparticles. The high viscosity of the high caseinate concentration possibly resulted in curcumin moving close to the hydrophobic part of the casein micelles, resulting in a lower encapsulation rate than those of low caseinate concentrations. Finally, the optimum parameters for preparing curcumin nanoparticles include a caseinate solution of 10 mg/mL and an ultrasound treatment time of 5 min.

Curcumin has been encapsulated into casein nanoparticles through several methods. The casein proteins self-assembled into spherical micelles with a mean size of about 166.3 nm. The curcumin molecules were attached to the casein micelles and produced complexes via hydrophobic bonding [15]. Native-like phosphocaseins with a mean diameter of 218 nm could provide a beneficial nanoscale system, and the curcumin–phosphocasein nanocomplexes were resistant to pepsin but not pancreatin digestion [16]. A hierarchical approach using the ligand binding of 1% curcumin to sodium caseinate is suitable to encapsulate curcumin and to form curcumin–casein nanoparticles with a mean diameter of 186.5 nm [19]. Compared with these methods, the proposed technique yielded nanoparticles of a smaller size (100–120 nm), which is close to the best size (50–100 nm) for cellular endocytosis [30].

### 3.3. Effect of Drug Loading on Stability of Curcumin–Casein Nanoparticles

**Figure 3:**
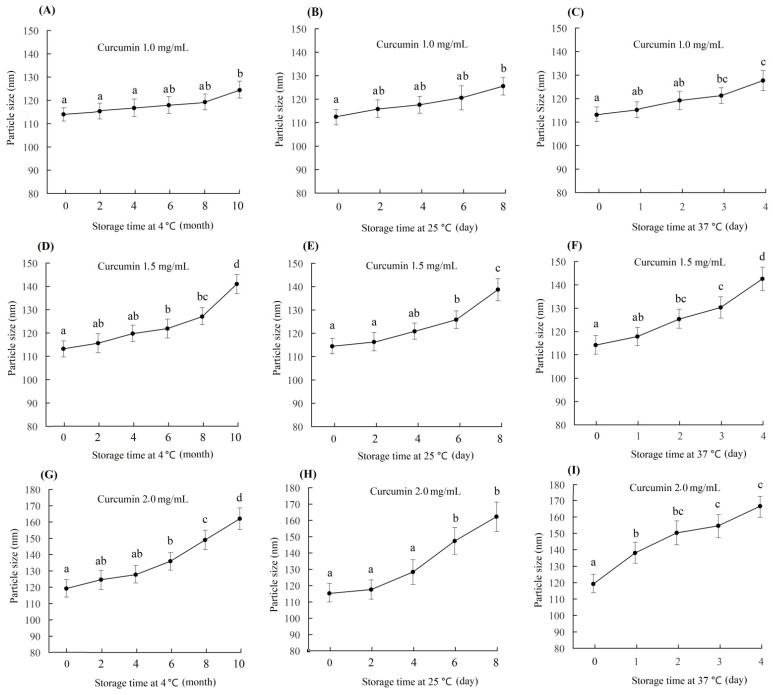
Change in curcumin–casein nanoparticle size during storage at 4 °C, 25 °C, and 37 ℃. The curcumin–casein nanoparticle dispersions were prepared with caseinate solution (10 mg/mL) and curcumin with curcumin/casein ratios of 1:10 (*m*/*m*) (**A**–**C**), 1.5:10 (*m*/*m*) (**D**–**F**), and 2:10 (*m*/*m*) (**G**–**I**). The values followed by a different lowercase letter are significantly different (*p* < 0.05).

In the preparation of curcumin–casein nanoparticles (Cur-CS-NPs), a high encapsulation rate could be achieved as the caseinate solution was 10 mg/mL and the curcumin/casein ratio reached 2:10 (*m*/*m*). The high drug loading rate may lead to instability in the nano-system. In this study, the particle size of three nano-systems prepared with a casein concentration of 10 mg/mL and curcumin/casein ratios of 1:10 (*m*/*m*), 1.5:10 (*m*/*m*), and 2:10 (*m*/*m*) were evaluated. Changes in the nanoparticle size during storage at a cold temperature (4 ℃), normal temperature (25 ℃), and body temperature 37 ℃ were checked for the three samples of curcumin nanoparticles with different drug loads. The particle size of the newly prepared Cur-CS-NPs was 110–120 nm, but the size significantly increased at a point of time during storage (Figure 3). Cur-CS-NPs prepared with a curcumin/casein ratio of 1:10 (*m*/*m*) were relatively stable under cold storage, and the particle size did not change significantly until the 10th month of storage. At room temperature, the particle size of Cur-CS-NPs did not change significantly within 6 days, but changed significantly after more than 2 days at body temperature. The particle size of Cur-CS-NPs prepared with a curcumin/casein ratio of 1.5:10 (*m*/*m*) began to increase significantly after 4 months of cold storage and 6 days at room temperature, whereas the particle size enhanced significantly on the second day of storage at body temperature. The particle size of Cur-CS-NPs prepared with a ratio of curcumin/casein of 2:10 (*m*/*m*) changed significantly after 4 months of cold storage and 4 days of storage at room temperature, but increased significantly after 1 day of storage at body temperature. The lower curcumin/casein ratio yielded more stable Cur-CS-NPs. When actually used, Cur-CS-NPs are required to be stable for different periods of time. We can select the appropriate drug load rate to prepare Cur-CS-NPs. For use in food, Cur-CS-NPs should be stable for 8 months in cold storage, 6 days at room temperature, and 2 days at body temperature in order to meet the requirements for available storage and oral delivery.

Through hydrophobic interactions, native casein micelles can attach to curcumin in an aqueous solution [31]. The curcumin concentration of the encapsulated curcumin dispersed in water was 136.7 μg/mL when the warm aqueous ethanol solution and sodium caseinate were spray dried, whereas the concentration only reached 30.2 ± 8.1 μg/mL when the curcumin was simply dissolved in the sodium casein solution. [17]. Our results show a high encapsulation level of curcumin, which yielded a dispersion of about 1.5 mg/mL which was achieved by pH-driven encapsulation [18]. This value is the highest loading amount reported in the literature. The stability of curcumin nanoparticles in its dispersion is a key property as the nanoparticles are used for the delivery of curcumin; the best size for cellular curcumin is about 50–100 nm. Ghayour et al. [19] reported that curcumin encapsulated in casein nanoparticles and prepared by continuous stirring was unstable based on the residue amount of curcumin during storage. The ultrasound method in our experiments led to good nanoparticle stability during storage.

### 3.4. Improving Effect of Curcumin–Casein Nanoparticles on the Inhibition of Arthritis In Vitro

**Figure 4:**
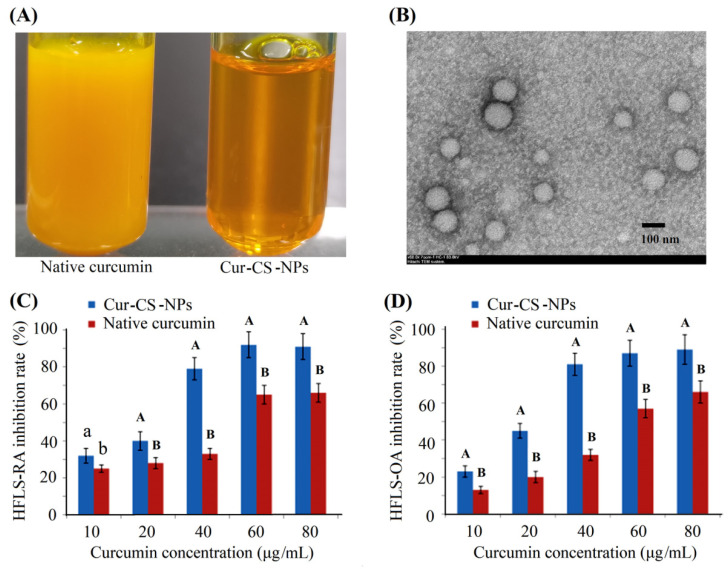
Photograph of native curcumin solution and Cur-CS-NPs dispersion (**A**), TEM photograph of curcumin-NPs (**B**), and inhibition activities against HFLS-RA cells (**C**) and HFLS-OA cells (**D**). The values over the bars of each drug dose group followed by different lowercase letters are significantly different (*p* < 0.05), and the values followed by a different capital letter are significantly different (*p* < 0.01). Cur-CS-NPs, curcumin–casein nanoparticles; TEM, transmission electron microscopy; NPs, nanoparticles; HFLS-RA, human fibroblast-like synoviocyte–rheumatoid arthritis cells; HFLS-OA, human fibroblast-like synoviocyte-osteo arthritis.

**Figure 5:**
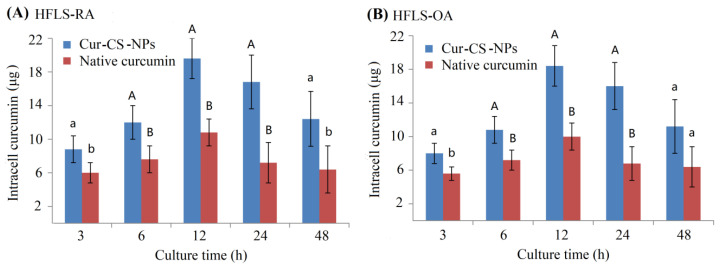
Curcumin amount in the intracells of HFLS-RA cells (**A**) and HFLS-OA cells (**B**) at 3 h, 6 h, 12 h, 24 h, and 48 h of culture when 40 μg curcumin was mixed into every well comprising 1 mL of the culture medium. Significant differences are shown by different lowercase letters over the bars in each group at *p* < 0.05, and significant differences are indicated by a lowercase letter and a different capital letter or by different capital letters at *p* < 0.01.

Curcumin has anti-arthritic effects for patients with osteoarthritis (OA) and rheumatoid arthritis (RA) [32,33]. We measured the inhibitory activity of Cur-CS-NPs on the proliferation of two kinds of arthritic cells compared with the native curcumin. Figure 4A shows Cur-CS-NP dispersion produced with curcumin and 10 mg/mL of caseinate with a curcumin/casein ratio of 1:10 (*m*/*m*). Native curcumin was obtained by dissolving curcumin in a mixed solution of polyethylene glycol (PEG300 (40%)-DMSO (10%)-Tween 80 (5%)-saline (45%) [34]. The nano-form performed better than the native curcumin in its ability to inhibit the proliferation of arthritic cells (Figure 4C,D), which possibly benefits from the availability of cellular uptake into Cur-CS-NPs (spherical with a diameter of about 50–120 nm) (Figure 4B), which is suitable for the endocytosis of cells [30]. Therefore, we performed a cellular uptake analysis for native curcumin and Cur-CS-NPs. The cellular uptake amount of curcumin for the nano-form was significantly larger than that of the native formulations at equivalent concentrations (Figure 5). This uptake profile explains the results of their inhibition activities against HFLS-RA cells and HFLS-RA cells. Hence, Cur-CS-NPs have the potential to deliver curcumin.

**Figure 6:**
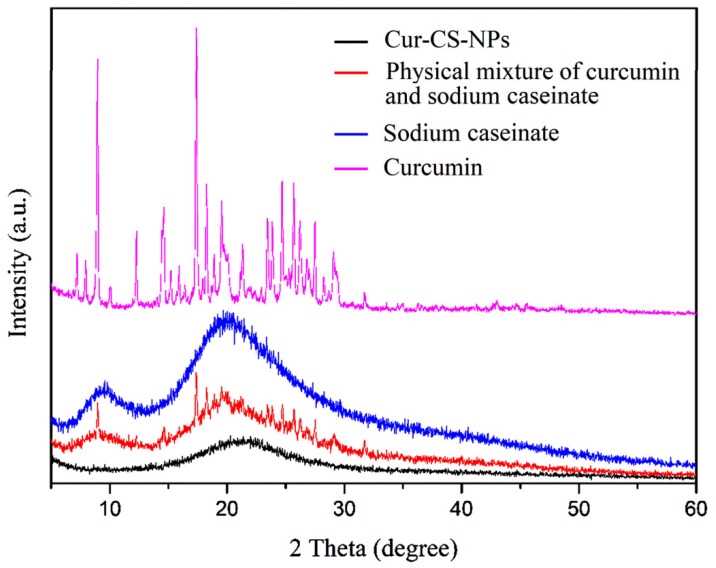
XRD patterns of curcumin (purplish red), sodium caseinate (blue), the physical mixture of curcumin and sodium caseinate (red), and Cur-CS-NPs (black). XRD, X-ray powder diffraction; a.u., arbitrary unit.

Curcumin has a low bioavailability due to its low water solubility and crystallinity. The Cur-CS-NP dispersion overcame the problem of low water solubility. In order to demonstrate the effect of crystallinity, we performed XRD of Cur-CS-NPs and compared them to the pristine. In contrast to sodium caseinate, which had the typical amorphous XRD pattern, pristine curcumin displayed multiple unique peaks due to its crystallinity (Figure 6). The distinct peaks of pristine curcumin vanished after encapsulation. In contrast, the simple physical mixture of pristine curcumin and sodium caseinate powder at the same ratio retained the specific peaks of pristine curcumin. The decline in crystallinity suggests that the encapsulated curcumin could be more effectively absorbed by the cells [16].

### 3.5. Inhibition Effect of Curcumin–Casein Nanoparticles on the Cytokines in Arthritic Cells

Arthritic cells can produce cytokines, namely IL-8, IL-1β, IL-6, and MMP-1, which are induced by TNF-α [27]. In the present study, the formation of inflammatory cytokines, namely IL-8, IL-1β, IL-6, and MMP-1, that were induced by TNF-α (20 ng/mL) was measured using ELISA after Cur-CS-NP and native curcumin treatment with a curcumin dose of 40 μg/mL. The results showed that the TNF-α-induced formation of IL-8, IL-1β, IL-6, and MMP-1 in HFLS-OA cells and HFLS-RA cells for the Cur-CS-NP formulation was lower than that observed for the native curcumin formulation (Table 1).

Even in the early stages of the disease, inflammation is thought to play a role in the formation and progression of OA. Secreted inflammatory substances, including proinflammatory cytokines, are important mediators of the altered metabolism and increased catabolism of the joint tissue associated with OA. The primary proinflammatory cytokines thought to be associated with the pathophysiology of OA contain TNF-α, IL-1β, and IL-6 [35]. In RA, a long-lasting autoimmune condition, the synovium accumulates cytokines and inflammatory cells which damage the joints. RA has various proinflammatory cytokines, such as IL-1, IL-6, IL-8, and TNF-α [36,37]. For RA patients, the inhibition of proinflammatory cytokine production is one of the most current therapies [38]. MMP-1 is essential for homeostasis in normal joints, but is elevated in RA [39]. In RA, MMP-1 and cytokines directly degrade bone and cartilage, which leads to joint degeneration. Based on the suppressed levels of inflammatory cytokines and MMP-1 in RA cells and OA cells, Cur-CS-NPs have a higher potential for the treatment of arthritis than native curcumin.

## 4. Conclusions

In this work, curcumin was encapsulated in casein micelles in order to form curcumin–caseins nanoparticles using an appropriate duration of ultrasound treatment, which drove the hydrophobic groups to the inner micelles and the polar sulfydryl groups to the surface of the micelles. The obtained curcumin–caseins nanoparticles had an appropriate size (100–120 nm) for cellular uptake and excellent stability, and exhibited significantly higher inhibitory activity against the proliferation and proinflammatory cytokines for both HFLS-OA cells and HFLS-RA cells compared to native curcumin. This was due to better cellular uptake resulting from the low crystallinity of curcumin in nano-form and the appropriate nano-size. The work time of ultrasound treatment obtained in the present study is the referential value for the incorporation of different drug molecules, and the curcumin–caseins nanoparticle form has the potential to treat arthritis.

## Figures and Tables

**Table 1:** Expression levels of cytokines in HFLS-RA cells and HFLS-OA Cells following TNF-α-induced formation after culture for 48 h *^a^*.

Group	MMP-1	IL-1β	IL-6	IL-8
HFLS-OA Cells				
Control	100 ^C *b*^	100 ^C^	100 ^C^	100 ^C^
Native curcumin	57.6 ± 4.1 ^B^	63.2 ± 5.4 ^B^	70.3 ± 5.9 ^B^	69.5 ± 5.2 ^B^
Cur-CS-NPs	42.4 ± 4.2 ^A^	37.1 ± 3.9 ^A^	39.1 ± 3.3 ^A^	33.1 ± 3.7 ^A^
HFLS-RA Cells				
Control	100 ^C^	100 ^C^	100 ^C^	100 ^C^
Native curcumin	56.8 ± 5.0 ^B^	66.2 ± 5.8 ^B^	62.3 ± 6.7 ^B^	59.5 ± 4.2 ^B^
Cur-CS-NPs	40.4 ± 3.1 ^A^	35.1 ± 2.9 ^A^	35.1 ± 3.7 ^A^	29.1 ± 3.2 ^A^

*^a^* The relative expression is shown as the % of the control group (100%). Mean ± standard deviation (SD), *n* = 3. *^b^* The values in a column with capital superscripts are significantly different (*p* < 0.01). HFLS-RA, human fibroblast-like synoviocyte–rheumatoid arthritis cells; HFLS-OA, human fibroblast-like synoviocyte-osteo arthritis; TNF-α, tumor necrosis factor α; Cur-CS-NPs, curcumin–casein nanoparticles; MMP-1, matrix metalloproteinase 1; IL-1β, interleukin-1β; IL-6, interleukin-6; IL-8, interleukin-8.

## Data Availability

Data is contained within the article.
